# Temporal Changes in Reverse Torque of Locking-Head Screws Used in the Locking Plate in Segmental Tibial Defect in Goat Model

**DOI:** 10.3389/fsurg.2021.637268

**Published:** 2021-04-27

**Authors:** Remigiusz M. Grzeskowiak, Rebecca E. Rifkin, Elizabeth G. Croy, Richard C. Steiner, Reza Seddighi, Pierre-Yves Mulon, Henry S. Adair, David E. Anderson

**Affiliations:** Department of Large Animal Clinical Sciences, College of Veterinary Medicine, The University of Tennessee, Knoxville, Knoxville, TN, United States

**Keywords:** animal models, segmental bone defect, bone healing, locking screws, orthopedics, biomechanics, fracture fixation

## Abstract

The objective of this study was to evaluate changes in peak reverse torque (PRT) of the locking head screws that occur over time. A locking plate construct, consisting of an 8-hole locking plate and 8 locking screws, was used to stabilize a tibia segmental bone defect in a goat model. PRT was measured after periods of 3, 6, 9, and 12 months of ambulation. PRT for each screw was determined during plate removal. Statistical analysis revealed that after 6 months of loading, locking screws placed in position no. 4 had significantly less PRT as compared with screws placed in position no. 5 (*p* < 0.05). There were no statistically significant differences in PRT between groups as a factor of time (*p* > 0.05). Intracortical fractures occurred during the placement of 151 out of 664 screws (22.7%) and were significantly more common in the screw positions closest to the osteotomy (positions 4 and 5, *p* < 0.05). Periosteal and endosteal bone reactions and locking screw backout occurred significantly more often in the proximal bone segments (*p* < 0.05). Screw backout significantly, negatively influenced the PRT of the screws placed in positions no. 3, 4, and 5 (*p* < 0.05). The locking plate-screw constructs provided stable fixation of 2.5-cm segmental tibia defects in a goat animal model for up to 12 months.

## Introduction

Animal models are commonly used in bone healing studies and the choice of animal models is vital to ensure valid results of the study (1–9). Selection of the species and type of model should be made accordingly to the research question, such as biocompatibility, osseointegration, bone healing, and bone regeneration. Important considerations for bone research include animal species, age, target bone, size of the defect, bone structure and vascularization, presence of periosteum, length of the study period, mechanical loads and stresses on the limb, and fixation methods (8, 9). Implants, such as bone fillers designated for use in orthopedics, should be tested in appropriate sites, such as the long bones, so that relevant biomechanical forces are exerted on the devices and that the device is exposed to the target environment (9).

Segmental bone defects of the tibia in goats have been well-established as a bone healing model in orthopedic translational studies (3–9). Several methods of defect stabilization have been described including external fixation (3, 4), dynamic compression plate (DCP) (5, 6, 10), limited contact dynamic compression plate (LC-DCP) (7, 11), and intramedullary nail (12). The appropriate fixation technique is essential for adequate bone stabilization as well as for a stable and consistent mechanical environment for healing bone (1, 8, 9). Locking plates are increasingly used to stabilize distinct fractures (13). They are particularly advantageous in the stabilization of non-load-sharing fractures such as comminuted bone fractures or bones with low bone mineral density (BMD) such as osteoporotic bones (13, 14). This plate system provides superior fixation stability when compared with conventional plating because the stability relies on the screw-plate interface and does not depend on the plate-bone interface to the same degree as conventional plating (15–17). Despite the advantages of these internal fixation devices, studies describing and evaluating the locking plates as the stabilization technique for segmental defects as well as looking at the long-term implant integration are lacking.

Peak reverse torque is a method commonly used in orthodontics to evaluate the osseointegration of the dental implants (18–20) as well it has been recently used in orthopedics to evaluate the stability and osseointegration of orthopedic implants under *ex-vivo* and *in-vivo* conditions (10, 21, 22). The reverse torque measures the torque required to break the bond between the screw, plate, and bone. This provides an indirect measurement of the screw-plate-bone interface integrity and it has been positively correlated with implant primary and secondary stability (10, 18, 22). Several factors have been described to significantly influence the primary and secondary implant stability, from which implant micromotion is most relevant (23). Cyclic axial compressive loading of the tibia defect model was recently shown under *ex-vivo* conditions to significantly reduce peak reverse torque of the screw implants placed within the locking plate (22). Evaluation of interface integrity of each screw placed in the locking plate provides insight into the entire construct biomechanics (10).

The first objective of this study is to report the long-term stability of the locking plate and osseointegration of the locking-head screws measured with the peak reverse torque at the time of plate removal. The second objective is to report the morbidities associated with the use of locking plate-screw constructs to stabilize the segmental bone defects, as assessed by radiographic imaging throughout 12 months of the study period. The study hypothesis was that the locking plate-screw constructs will provide stable fixation of non-load sharing bone defects and that the stability of the constructs will change over time as a result of cyclic axial loading.

## Materials and Methods

### Defect Creation Surgery

**Figure 1:**
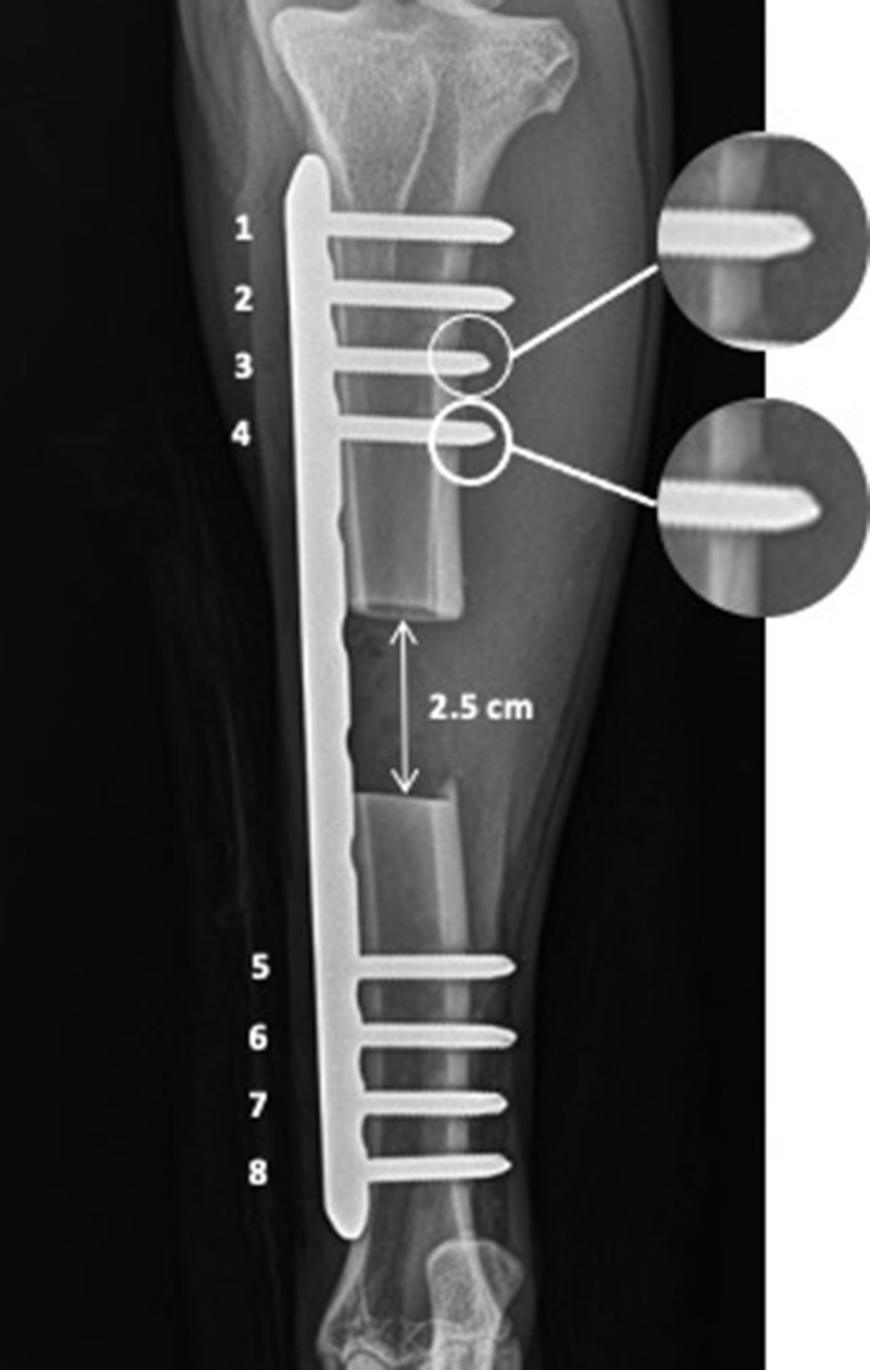
The 2.5 cm osteotomy was stabilized with 8-hole, 4.5 mm thick locking plate and eight 4.0 mm locking-head screws, four in the proximal bone segment in positions from 1 to 4 and four in the distal bone segment in the positions from 5 to 8. The transcortical diaphyseal fractures are marked with the white circle and enlarged on the right side of the image.

The goats involved in this study were part of an orthopedic research project assessing bone healing over 12 months. The study was conducted under a protocol (#2382-1215) approved by the Institutional Animal Care and Use Committee of the University of Tennessee. Each goat had a 2.5-cm long segmental tibia defect created in the mid-diaphysis of the bone. Each of the segmental tibia defects was stabilized with a custom-designed low contact round double threaded 8-hole, 4.5-mm thick locking plates^1^ with a solid central portion between the screw holes. The plate was stabilized against the bone using eight 4.0-mm diameter locking-head self-tapping screws^2^ (Figure 1). Four screws were placed in the proximal bone segment in positions #1, #2, #3, and #4; four screws were placed in the distal bone segment in positions #5, #6, #7, and #8 as previously described (Figure 1) (22). All screws were self-tapping and placed according to the AO/ASIF guidelines including pre-drilling a pilot hole, low-speed drilling, and continuous saline lavage to prevent excessive heat generation during drilling.

The segmental defect creation surgeries were performed in the following fashion. After placement of the jugular vein polyurethane 16GA × 7.5 cm catheter^3^, goats received perioperative antibiotics that included Ceftiofur Sodium^4^ (6.6 mg/kg IV, q12h) and non-steroidal anti-inflammatories (NSAIDs) that included Flunixin Meglumine^5^ (1.1 mg/kg IV, q12h). Goats were sedated with Xylazine^6^ (0.05 mg/kg IV). General anesthesia was then induced using a mixture of Ketamine Hydrochloride^7^ (5 mg/kg IV) and Midazolam^8^ (0.5 mg/kg IV), titrated to effect. After tracheal intubation, general anesthesia was maintained and adjusted to the desired depth, using Isoflurane^9^ (1–2 vol%) in oxygen (1–2 L/min). Goats were placed in dorsal recumbency and the right hind limb was suspended in an extended position. The right hindlimb was clipped from the mid-femur to the mid-metatarsus, aseptically prepped, and draped accordingly for surgery. An ~20-cm long incision was made over the medial aspect of the tibia and the periosteum was stripped away from the surface of the bone using periosteal elevators^10^. The distance from immediately proximal to the medial malleolus to a point distal to the medial condyle of the tibia was spanned using the eight-hole 4.5 mm locking plates (length range, 14–18-cm). The plate was applied to the cranial medial aspect of the tibia and stabilized with locking screws. All screws were placed in the same fashion: first, a 3.2 mm diameter locking-head drill sleeve^11^ was secured in the holes of the locking plate; then, a guide hole was drilled using battery-powered orthopedic drills^12^ and a 3.2-mm diameter drill bit^13^. The drill bit and screw hole were lavaged with sterile normal saline to cool the bit and clear the remaining debris. A depth gauge^14^ was used to measure the desired length of the screw. Each self-tapping screw was inserted using a hand-held screwdriver^15^. The locking-head self-tapping screws were manually driven into the bone until the cutting flute (2–3 threads of the screw) extended beyond the far cortex.

After initial placement of screws in positions #1 and #8, a full-thickness, the 2.5-cm segmental defect was created in the center of the bone using an oscillating bone saw^16^. Following defect creation, the screws adjacent to the osteotomy (positions #4 and #5) were placed and the remaining four screws were placed in the screw positions (#2, #3, #6, and #7). All screws were hand tightened until each screw was tight. Soft tissues were closed in a routine fashion and the goats recovered from anesthesia. Full limb bandages with two, one medial and one lateral plastic splints^17^ (length, 30.5 cm) were applied before recovery. The length of the splints spanned the hind limbs from the foot to stifle. After surgery, the goats were continued on postoperative antibiotics that included Ceftiofur Sodium (see text footnote 4) (6.6 mg/kg IV, q12h) and NSAIDs that included Flunixin Meglumine (see text footnote 5) (1.1 mg/kg IV, q12h) for 3 days. If any goat required extended pain management due to increased lameness, it was administered Meloxicam^18^ (0.5 mg/kg PO, q24h) for additional 3 days.

### Postoperative Monitoring and Peak Reverse Torque Measurements

The study timeline included four time points, including groups at 3 months (*n* = 16), 6 months (*n* = 24), 9 months (*n* = 23), and 12 months (*n* = 20). Goats that had postoperative complications such as surgical site infection, fracture of proximal or distal bone aspect were excluded from further analysis. Goats were housed in individual stalls (1 × 3 m) for up to 30 days after the surgery before returning to group housing pens of up to six goats per pen (3 × 6 m). Full-limb splint bandages were removed within 60 days after the surgery. Goats were housed in group pens for up to 6 months after surgery and then turned out into grass paddocks until individual study endpoints had been reached. The stability of the locking plate-screw constructs was assessed monthly by radiographic imaging^19^. Two orthogonal radiographic projections were done at each time point using craniocaudal and mediolateral projection. Goats were euthanized^20^ at 3, 6, 9, and 12 months after surgery, and peak reverse torque (PRT) was measured before screw removal. PRT was measured using a digital hand-held torque-measuring screwdriver^21^ at the time of screw and plate removal. Peak reverse torque was measured starting from the most proximal screw position (#1) to the most distal screw placed in position (#8). PRT was measured during the first 25 degrees of rotation with the screwdriver. Real-time continuous data acquisition was conducted using software^22^ compatible with the screwdriver.

### Data Acquisition and Organization

The results of peak reverse torque measurements were imported into Excel spreadsheets (see text footnote 22). Radiograph images were assessed, and stability of locking plate-screw constructs evaluated (position, presence of intracortical fractures). Radiographs were taken within 24 h after the surgery were evaluated for screw associated intracortical fractures at each site of screw penetration (cis- and trans-cortices) occurrence. All radiographs were evaluated for morbidities including plate displacement relative to the proximal or distal bone segments, plate bending, or screw backout. The proximal and distal bone positions were further evaluated for the presence of osteolysis and periosteal and/or endosteal reaction at both cis- and trans-cortices.

### Statistical Analysis

The statistical analysis was conducted using the SPSS software^23^ and the power of the study was calculated with the PS software^24^. Descriptive statistics, including the mean, standard deviation, and percentage were calculated and presented. The data in all variables was analyzed for normality of distribution using the Kolmogorov-Smirnov test. For data not normally distributed, the analysis was accomplished using non-parametric statistical tests. The mean PRT values for each screw position were compared among all the study endpoints (from 3 to 12 months) using Kruskal-Wallis and Mann-Whitney tests with Bonferroni correction for multiple comparisons. The mean PRT values were further compared within the same study endpoints among the screw positions with Kruskal-Wallis and Mann-Whitney tests with Bonferroni correction for multiple comparisons.

The 2-tailed Spearman correlation test was used to analyze the influence of the weight of each goat and time after surgery on the mean PRT values as well as the influence of morbidity factors such as the presence of screw associated intracortical fractures and factors which had occurred in the following postoperative period such as plate displacement, plate bending, screw back out, presence of osteolysis and periosteal and/or endosteal reaction. The correlation of screw associated intracortical fractures and screw backout occurrence relative to screw positions was analyzed with the Chi-square test. The correlation of osteolysis, periosteal, and/or endosteal reaction presence with the proximal and distal bone segment was analyzed with the Chi-square test. Statistical significance was established at *p* < 0.05. Accounting for the difference in PRT results between the time treatment groups found in this study and methods of statistical analysis, the power of the study to detect the true difference was calculated to be ß > 0.9.

## Results

The surgeries were conducted on 96 goats and 13 goats were excluded from further analysis. Twelve goats were excluded due to the surgical site infection and one because of the fracture of proximal bone segment. PRT data was measured on 664 locking-head screws in 83 skeletally mature female goats (age range, 2–6 years). Based on pre-assigned study endpoints, PRT measurements were obtained from 16 goats 3 months after the surgery, 24 goats at 6 months, 23 goats at 9 months, and 20 goats 12 months after surgery. The length of the screws used to fix the locking plate to bone ranged between 28 and 36 mm. The mean weight of goats included in 3-, 6-, 9-, and 12-months treatment group equaled 51.50 ± 7.2 kg, 51.50 ± 5.8 kg, 54.91 ± 7.1 kg, and 56.45 ± 7.2 kg respectively. The mean PRT measured 3, 6, 9 and 12 months after surgery equaled 195.78 ± 32.9 Ncm, 158.48 ± 52.9 Ncm, 153.48 ± 24.0 Ncm, and 134.69 ± 17.1 Ncm, respectively; no statistically significant differences were found between the treatment groups as well no statistically significant correlation was found between PRT and time (*p* > 0.05; Table 1). The weight of the goats was not correlated with the mean PRT (*p* > 0.05). Analysis of the mean PRT of screws placed in each screw position revealed that at 6 months after surgery, the mean PRT of screws placed in position #4 were significantly less than the mean PRT of the screws placed in position #5 (*p* < 0.05; Table 1). No other significant differences between other screw positions were found (*p* > 0.05; Table 1).

**Table 1:** Peak Reverse Torque (PRT) measurements.

**Screw Position**	**3 Months**	**6 Months**	**9 Months**	**12 Months**
1	97.3 (5.8–684.8)^a, α^	165.7 (7.5–622.6)^a, α^	113.7 (6.0–557.9)^a, α^	56.6 (3.2–484.9)^a, α^
2	146.8 (37.4–567.3)^a, α^	130.9 (5.1–506.1)^a, α^	68.6 (4.2–802.6)^a, α^	105.1 (2.5–429.9)^a, α^
3	87.6 (6.3–537.8)^a, α^	86.1 (5.4–445.8)^a, α^	86.7 (9.1–802.6)^a, α^	108.8 (3.9–270.3)^a, α^
4	102.8 (9.7–435.4)^a, α^	67.7 (2.5–473.7)^a^,	91.5 (4.2–449.3)^a, α^	117.2 (6.1–233.2)^a, α^
**Combined (1–4)**	**100.5 (5.8–684.8)**	**89.4 (2.5–622.6)**	**87.2 (4.2–802.6)**	**105.6 (2.5–484.9)**
5	198.4 (50.5–563.4)^a, α^	170.9 (19.3–541.1)^a, γ^	182.1 (49.2–430.6)^a, α^	116.7 (44.7–369.9)^a, α^
6	142.2 (48.3–451.3)^a, α^	85.8 (21.6–399.4)^a, α^	85.7 (18.3–443.0)^a, α^	76.0 (21.9–319.7) ^a, α^
7	90.2 (28.9–802.7)^a, α^	77.4 (14.3–599.7)^a, α^	93.7 (23.2–331.9)^a, α^	116.6 (6.7–346.3)^a, α^
8	127.3 (32.1–656.4)^a, α^	99.8 (17.9–548.2)^a, α^	98.2 (23.2–331.9)^a, α^	139.8 (3.4–409.2)^a, α^
**Combined (5–8)**	**127.3 (28.9–802.7)**	**104.6 (14.3–599.7)**	**106.6 (18.3–443.0)**	**108.6 (3.4–409.2)**
**Median**	**115.0**	**92.9**	**92.6**	**112.7**

*Statistically significant difference between the treatment groups for the same screw position were labeled with different alphabetic letters and statistically significant difference between screw positions within the same treatment groups were labeled using different Greek letters. The median PRT values of combined screw positions (1 to 4 and 5 to 8) as indicated in the left column are bold. The median PRT value of all combined screws positions as indicated in the left column is also bold*.

Analysis of radiographs taken immediately after surgery revealed that intracortical fractures occurred in the far cortex (trans-cortex) associated with screw placement in 151 out of 664 screws (22.7%) (Table 2). These fractures occurred significantly more often in screws placed adjacent to the osteotomy (*p* < 0.05; Table 2). Radiographs taken at each study endpoint found few screws associated intracortical fractures. Presence of these fractures immediately after the surgery or at the termination time point were not significantly correlated with the mean PRT at any of the study endpoints (*p* > 0.05).

**Table 2:** Transcortical diaphyseal tibial fractures and backout screws occurrence.

**Screw position**	**Transcortical diaphyseal fractures**		**Backout screws**	
	**No. of screws**	**% of total screws**	**No. of screws**	**% of total screws**
1	8^a^	9.6	6^b^	7.2
2	15^a^	18.1	8^b^	9.6
3	25^b^	30.1	8^b^	9.6
4	26^b^	31.3	8^b^	9.6
5	29^b^	34.9	1^a^	1.2
6	22^b^	26.5	2^a^	2.4
7	16^a^	19.3	2^a^	2.4
8	10^a^	12.0	1^a^	1.2
Total	151	22.7	36	5.4

*Statistically significant difference in the number of affected screw positions between the screw positions and bone segments was labeled with different alphabetic letters*.

Further analysis of monthly radiographs revealed that the complete defect bridging with the new bone was observed at 3, 6, 9, and 12 months after the surgery in 43.8, 58.3, 65.2, and 90.0% of the goats assigned to each study endpoint, respectively. Complete defect bridging was not significantly correlated with the mean PRT at any of the study endpoints (*p* > 0.05). The locking plate-screw constructs partially displaced from the proximal bone segment in six out of 83 goats (7.2%). Plate partial displacement from the distal bone segment was not observed. Bending of the locking plate-screw constructs occurred in eight out of 83 goats (9.6%). Bending occurred in the center of the plate in the region where the segmental defect had been created, between the screws adjacent to the osteotomy (position #4 and #5). Neither partial displacement of the plate (*p* > 0.05) nor plate bending (*p* > 0.05) was significantly correlated with the mean PRT. At each study endpoint, complete bone bridging was observed in two out of 6 goats (33.3%) that presented partial plate displacement and in four out eight goats (50.0%) that presented plate bending. Periosteal and endosteal reaction in the cis- and/or trans-cortex occurred significantly more often in the proximal bone segments [40 (48.2%) out of 83 goats] as compared with the distal bone segments [17 (20.5%) out of 83 goats] (*p* < 0.05). The excessive periosteal and endosteal reaction was not significantly correlated with mean PRT (*p* > 0.05). Screw loosening and backout occurred significantly more often in the proximal bone segments [30 (9.0%) out of 332 proximal screws] as compared with distal bone segments [6 (1.8 %) out of 332 distal screws] (*p* < 0.05). Screw loosening and back out of screws placed in positions #1, #2, #3, and #4 had a significant negative influence on the mean PRT of the screws placed in positions no. 1, 3, and 4 (*p* < 0.05; Table 2). The screws back out occurred more often in the 3-, 9-, and 12-months treatment groups with 11 screws, 14 screws, and 11 screws backing out, respectively (*p* < 0.05). None of the screws backed out within the 6-months treatment group.

## Discussion

Locking plate-screw constructs provided stable fixation based on the results of this study. The frequency of clinically significant complications encountered during the study was low and did not require removal of the affected goats from the study. Locking plates with a solid section spanning bone defects, are suitable for use in this type of defects stabilization. Fixation with locking plates may offer advantages compared with conventional plates because it does not rely on plate and bone contact as extensively (15–17). The stability of the fixation provided by the locking plates is granted by the cumulative strength of each screw and plate interface (15–17). Limb loading results in forces being entirely transmitted to the implants because the segmental defect model requires that the screw plate construct fully shields the created defect from loading, providing a biomechanical stabilization of the limb (15–17). The advantage of these constructs makes the locking plates desirable to stabilize these segmental bone defects. An additional advantage over other constructs is that this allows for long-term stabilization, a particular advantage compared with external skeletal fixation, and preserves the bone gap, without obstruction, for the study of bone fillers and other devices, a particular advantage compare with intramedullary nails.

The osseointegration of the screws, associated with stability provided by the screw-plate construct, was evaluated using PRT measurements. The obtained PRT values were similar among all the screw positions within the various time points after surgery. This supports a consistent post-screw insertion primary stability and the establishment and persistence of secondary stability for these screws. There was no statistically significant differences in PRT between the treatment groups, however there was a PRT reduction trend over time after surgery. Weakening of the screw-plate and screw-bone interface strength could have resulted from shielding from loading stress in bone adjacent to the screw implants or low-grade infection (24–26). Since loading is directly transmitted into forces experienced by the locking plate-screw constructs, bone adjacent to the screws is shielded from loading and therefore is expected to undergo resorption over time, weakening the screw-bone interface (26–29). In this study, this was more pronounced within the proximal bone fragments as compared with the distal bone fragments. The difference in the strength of screw-bone interface has been proposed to be associated with non-uniform distribution of loading within plate-screw-bone constructs (10, 22, 30). In a previous study, we reported PRT for screws placed in dynamic compression plates (DCP) used to stabilize segmental bone defects for a period of 60 days (10). A significant difference was found for PRT between the screws placed proximal to the osteotomy and those placed distal to it (10). Screws placed in the proximal segment obtained significantly less PRT than screws placed distal to the osteotomy (10). The difference in strength of screw-bone interfaces after 60 days of *in-vivo* loading between the proximal and distal bone was explained with greater loading experienced by the proximal bone fragment and greater bone mineral density (BMD) in the distal bone segment (10). Greater BMD has been associated with a greater bone to implant contact (BIC) which has been further significantly positively correlated with osseointegration (19, 20, 31).

The PRT values in this study were greater as compared with another study using this method to evaluate the osseointegration of screws placed in a dynamic compression plate (DCP) (10). The mean PRT of the screws placed within DCP was 66 Ncm after 2 months of *in vivo* loading (10) as opposed to this study in which mean PRT ranged from 135 to 196 Ncm. Greater PRT values suggest that the screw-plate interface may contribute to the stability of the screws in locking plates and may result in improved osseointegration of the construct compared to the conventional plating.

Complications associated with locking plate fixation are not uncommon. Locking plates are known to provide a stable, stiff fixation (23, 32, 33). These constructs create single beam constructs which are defined with no motion between the single elements of the beam, including plate, screws and bone (15, 16). Single beam constructs have been found to be four times stronger than conventional plating (15). Locking plates warrant a fixed-angle stabilization with the screws placed perpendicular to the plate (15). Compression forces applied to the screws effect the bending stress within the plate subjecting the construct to cyclic fatigue (15, 22, 34). The fatigue of the construct may result in bending of the implant. The mechanism explaining plate-screw construct displacement away from the proximal bone fragment is unknown, however, it is expected that this is associated with cyclic fatigue of the plate-screw construct and resulting failure of the screw-bone interface, possibly associated with less mineral density in the bone immediately surrounding the screws. It is also possible that repetitive stress applied to the screw-bone interfaces leads to a damage of those interfaces weakening their strength. Proximal aspect of the human and equine tibia has been also shown to experience relevant amount of torsional forces that could have contributed to the failure of the screw-bone interface (35, 36). During the screw placement, overtightening of screws can influence construct stabilization and lead to plate and screws backout (37). This should be less of a concern with locking plates as the screw tightens against the screw-plate interface predominantly. Periosteal and endosteal reactions observed more commonly in the proximal bone segment could have been related to reduced fixation stability of the proximal bone segment or to a stress shielding created by the angle-stable locking plate (23).

The mechanism of screws loosening resulting in back out from the construct, and, likewise, the mechanism of plate and screws displacement away from the bone, is unclear. A greater number of screws backed out within the proximal bone segment as compared with the distal bone fragment. This also could be associated with cyclic fatigue of the construct, greater loads experienced by the proximal screws, torsional forces and lesser bone density in the proximal tibia (10, 11, 22, 35, 36). Once the screw-locking plate interface, or the screw-bone interface has been disrupted, compressive forces applied to the screws can cause rotational micromotion of the implant impairing osseointegration and weakening the screw-bone interface (21–23). The weakened screw-interface would allow movement in the direction of least resistance with continuous rotational micromotion gradually leading to the implant backing out from the plate (21–23). A recently published study found that the torque required to remove the locking-head screws from the locking plates used to stabilize non-load-sharing bone defects is significantly reduced already after 5000 cycles of axial loading (22). Since the osseointegration starts within 10–14 days after implantation, excessive micromotion of the proximal screws could have impaired their osseointegration (38). The reason why none of the screws backed out after 6 months after surgery is unknown. During the internal fixation procedures, after all implants had been placed, the screws need to be tightened at the end of the procedure to ensure primary stability has been achieved. Several studies have shown a difference between the torque exhibited by different surgeons (39, 40). However, insertional torque in locking plates is related to the screw and plate interface and satisfactory torque is achieved when the screw head fully engages the plate and can no longer be advanced.

Screw associated intracortical fractures have been previously described as a common incidence during screw placement (10, 41, 42). These fractures have been found to be positively correlated with insertional torque and, therefore, their incidence has been reported to be greater when using self-tapping screws as compared with tapped (non-self-tapping) screws (41, 42). In this study, intracortical fractures healed within the study periods based on analysis of radiographs. In a previous study, we did not find any correlation between the occurrence of microfractures and PRT (10).

This study had several limitations. The most important limitation was associated with the lack of measurement of insertional torque or use of a torque limiting screwdriver. Screws were also placed by different surgeons; as a result, insertional torque could not be standardized. Distinct values of the insertional torque could have influenced primary and secondary stability of the screw implants. During the surgery, a care was taken that all the screw heads were fully engaged in the locking plate holes, reducing therefore the risk of excessive micromotion (reduced primary stability) in the postoperative period. The utilization of a torque limited screwdriver would have allowed for the standardized screw placement and reduced the range of the results. Further, the attempts to measure bone mineral density (BMD) were not performed. BMD has been previously positively associated with the reverse torque and it would have been beneficial to standardize bone quality of the goats used in this study. BMD could have been measured with dual x-ray absorptiometry (DEXA) or quantitative computed tomography (qCT). Lack of BMD measurement was addressed by limiting the variability in the goat population. All goats included in the study had a similar weight, similar age, were intact females, and were free from any other orthopedic conditions.

## Conclusion

The locking plates provided a stable fixation for segmental tibia defects in goats for up to 12 months. The relatively low number of complications confirms that locking plates were suitable implants for the stabilization of segmental tibial defects. Similar strength of the screw-plate and screw-bone interfaces among the screw positions resulted in rigid fixation of the defect. The trend of reducing PRT over time after surgery could have been associated with the stress shielding mechanism as well as with negative effect of screw implant micromotion caused by the cyclic loading as it was shown under *ex-vivo* conditions.

## Data Availability Statement

The original contributions presented in the study are included in the article/Supplementary Material, further inquiries can be directed to the corresponding author/s.

## Ethics Statement

The animal study was reviewed and approved by University of Tennessee Institutional Animal Care and Use Committee (IACUC).

## Author Contributions

RG performed surgical procedures, reverse torque measurements, analyzed the radiographs, organized, and analyzed data as well as wrote the manuscript. RR performed surgical procedures, reverse torque measurements, and assisted with data organization. EC assisted with surgical procedures, reverse torque measurements, analyzed the radiographs, and assisted with data organization. RCS assisted with surgical procedures, reverse torque measurements, and anesthesia. RS performed the anesthesia and monitoring during surgical procedures as well as supervised manuscript writing. P-YM performed surgical procedures as well as supervised data collection, analysis, and manuscript writing. HA performed surgical procedures as well as supervised data collection, analysis, and manuscript writing. DA mentored the project, performed surgical procedures, supervised data collection, and analysis as well as manuscript writing. All authors have read and approved the final submitted manuscript.

## Conflict of Interest

The authors declare that the research was conducted in the absence of any commercial or financial relationships that could be construed as a potential conflict of interest.

## Footnotes

^1^316 Stainless Steel Orthopedic Locking Plates, Veterinary Orthopedic Implants, St. Augustine, FL.

^2^316 Stainless Steel Locking-head Screws, Veterinary Orthopedic Implants, St. Augustine, FL.

^3^Extended use MILACATH®, MILA international Inc., Florence, KY.

^4^Naxcel®, 4g powder, Zoetis®, Parsippany, NJ.

^5^Banamine, 50 mg/ml, Merck®, Kenilworth, NJ.

^6^AnaSed® 20 mg/ml, VetOne®, MWI Animal Health, Boise, ID.

^7^Zetamine® 100 mg/ml, VetOne®, MWI Animal Health, Boise, ID.

^8^Midazolam, 5mg/ml West-Ward Pharmaceuticals, Eatontown, NJ.

^9^Isoflurane, VetOne®, MWI Animal Health, Boise, ID.

^10^316 Stainless Steel Periosteal Elevator, Veterinary Orthopedic Implants, St. Augustine, FL.

^11^316 Stainless Steel Drill Sleeve, Veterinary Orthopedic Implants, St. Augustine, FL.

^12^Model Number: ND-1001, Anhui, China.

^13^316 Stainless Steel Drill Bit, Veterinary Orthopedic Implants, St. Augustine, FL.

^14^316 Stainless Steel Depth Gauge, Veterinary Orthopedic Implants, St. Augustine, FL.

^15^STAR 4.0 screwdriver, Veterinary Orthopedic Implants, St. Augustine, FL.

^16^Power Oscillating Bone Saw, DEJUN, Shenzhen, China.

^17^Hind leg splint set, medium, Premier1Supplies, Washington, IA.

^18^Meloxicam, 15 mg tablets, Unichem Pharmaceuticals Inc., East Brunswick, NJ.

^19^NEXT Equine DR™, Sound Technologies, Carlsbad, CA.

^20^Euthasol 100 ml, Virbac, Westlake, TX.

^21^Cedar DID-4 Digital Torque Screwdriver, Sugisaki Meter CO, Ibaraki, Japan.

^22^Microsoft Office Professional Plus 2016, Microsoft, Redmond, WA.

^23^IBM SPSS v. 26, New York, NY.

^24^PS power and sample size analysis software v. 3.0, 2009.

## Abbreviations

AO/ASIF: Arbeitsgemeinshaft Osteosynthesefragen / Association for the Study of Internal Fixation
BIC: Bone-Implant Contact
BMD: Bone Mineral Density
DCP: Dynamic Compression Plate
DEXA: Dual X-ray Absorptiometry
LC-DCP: Limited Contact Dynamic Compression Plate
NSAIDs: Non-steroidal Anti-inflammatories drugs
PRT: Peak Reverse Torque
qCT: Quantitative Computed Tomography.
